# When the victim becomes the villain: Platelets as drivers of immune dysregulation in ITP

**DOI:** 10.1016/j.jtauto.2025.100309

**Published:** 2025-08-19

**Authors:** Fatemeh Farhid, Hadi Rezaeeyan, Reza Habibi, Ehsan Kamali Yazdi, Michael R. Hamblin, Jalal Naghinezhad

**Affiliations:** a Blood Transfusion Research Center, High Institute for Research and Education in Transfusion Medicine, Iranian Blood Transfusion Organization Building, Next to the Milad Tower, Hemmat Exp. Way, P.O.Box:14665-1157, Tehran, Iran; b Tehran University of Medical Science, Tehran, Iran; c Alborz University of Medical Sciences, Alborz, Iran; d Laser Research Centre, University of Johannesburg, Doornfontein, 2028, South Africa; e Asadabad School of Medical Sciences, Asadabad, Iran

**Keywords:** Immune thrombocytopenia, Platelet activation, P-Selectin, P-Selectin glycoprotein ligand 1, Transforming growth factor β, Immune dysregulation, Hairy platelets

## Abstract

Immune thrombocytopenia (ITP) is a heterogeneous autoimmune disorder characterized by immune-mediated destruction of platelets and impaired platelet production. Although autoantibodies have historically been central to the understanding of ITP, current evidence demonstrates that its pathogenesis extends well beyond humoral mechanisms to involve complex dysregulation of both innate and adaptive immune responses. Multiple immune pathways—including autoreactive B and T cells, dendritic cell activation, and regulatory T cell deficiency—contribute to disease onset, progression, and chronicity. Moreover, ITP encompasses a broad spectrum of clinical and immunological subtypes, including primary idiopathic forms and secondary ITP associated with autoimmune diseases, infections, and inborn errors of immunity. This review offers a novel perspective on ITP pathogenesis, emphasizing the active immunoregulatory role of platelets as contributors to immune dysregulation. Far from being passive targets, platelets in ITP actively shape immune responses through crosstalk with immune cells, particularly CD4^+^ T helper (Th) and CD8^+^ cytotoxic T cells. This interaction, primarily mediated via the P-selectin–PSGL-1 axis, promotes Th1/Th17 polarization, enhances autoantibody production, and accelerates platelet destruction. In parallel, platelet-derived microparticles (PMPs) act as potent immune effectors by delivering pro-inflammatory cytokines and autoantigens that sustain chronic immune activation. Prolonged platelet activation also gives rise to a distinct subpopulation known as “hairy platelets”—exhausted, granule-depleted cells with altered surface phenotypes and sustained pro-inflammatory potential. Although functionally exhausted in terms of coagulation, these platelets retain immunostimulatory capacity through persistent phosphatidylserine exposure and cytokine release. By reframing platelets as active participants in the pathogenesis of ITP, this review proposes that targeting platelet activation, platelet–T cell interactions, and PMP release may represent innovative therapeutic strategies. Such approaches could offer more precise and personalized treatment options, particularly for patients with chronic or refractory disease, by restoring immune balance and improving long-term outcomes.

## Highlights

• **Activated platelets in ITP act as potent pro-inflammatory effectors**, amplifying immune dysregulation and driving chronic inflammation.
• **Procoagulant platelets**—functionally “**exhausted**” and sometimes morphologically “**hairy**”—are granule-depleted, **PS**-**exposing** cells that release microparticles, fueling immune dysfunction and inflammatory cascades.
• Platelet–T cell interactions via the **P-selectin–PSGL-1** axis activate T helper and cytotoxic T cells, perpetuating autoimmune responses and accelerating platelet destruction.
• **Platelet-derived microparticles** carry pro-inflammatory cytokines and autoantigens, sustaining chronic immune activation and promoting autoreactive immune responses.
• Therapeutically targeting platelet activation, **platelet–T cell interactions**, and platelet microparticle release offers a precise strategy for managing chronic and refractory ITP, with the potential to improve patient outcomes.

## Graphical abstract

**Figure:**
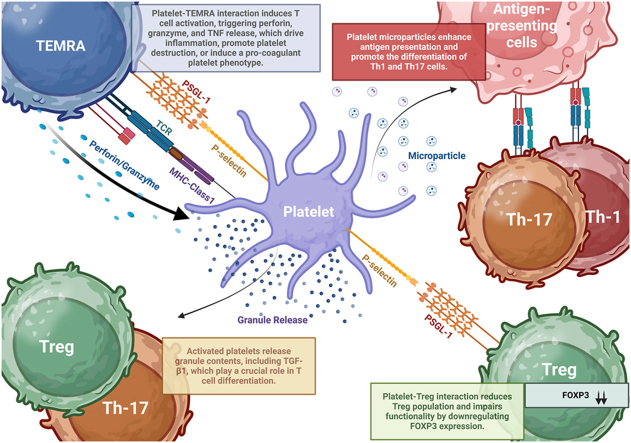

## 1 Introduction

Immune thrombocytopenia (ITP) is a complex and heterogeneous autoimmune disorder characterized by both increased platelet destruction and impaired platelet production [1]. While it has traditionally been described as primarily driven by anti-platelet autoantibodies [2], it is now clear that the pathogenesis of ITP extends beyond humoral immunity to involve cellular immune mechanisms. Aberrant T and B cell responses, antigen-presenting cell activation, and regulatory T cell (Treg) deficiency all contribute to the disruption of immune tolerance and the perpetuation of disease [3]. Additionally, ITP manifests as a spectrum of clinical presentations, from acute, self-limiting cases in children to chronic, refractory forms in adults, and can be classified as either primary idiopathic or secondary to other autoimmune diseases, infections, or inborn errors of immunity [[4], [5], [6]].

While our understanding of ITP's immunopathogenesis has evolved, platelets are still often portrayed as passive targets of immune attack. Recent findings in platelet biology challenge this perspective, suggesting that platelets themselves are active participants in immune regulation [7,8]. These insights form the basis of this review, in which we propose a shift in the pathogenic paradigm: rather than being mere victims of immune-mediated destruction, platelets may actively contribute to the chronic immune imbalance characteristic of ITP.

Emerging evidence highlights the central role of platelet activation in modulating immune responses. Activated platelets dynamically interact with immune cells, including CD4^+^ T helper (Th) and CD8^+^ cytotoxic T lymphocytes [7,9,10]. Through mechanisms such as the P-selectin–PSGL-1 axis, platelets form stable aggregates with T cells, enhancing their activation and prolonging inflammatory responses [11]. Additionally, platelet expression of major histocompatibility complex class I (MHC-I) enables direct engagement with T-cell receptors (TCRs), promoting the expansion of effector memory CD8^+^ T cells (TEMRA) and driving Th1/Th17 polarization—key features of autoimmune progression in ITP [9].

Beyond cell–cell interactions, platelet-derived microparticles (PMPs) serve as potent mediators of inflammation. These vesicles carry pro-inflammatory cytokines, chemokines, and platelet autoantigens, creating a self-perpetuating loop of immune activation and thrombocytopenia [12,13]. Chronic platelet activation can further generate dysfunctional, procoagulant platelets—morphologically described as “hairy” due to their surface projections—characterized by granule depletion and phosphatidylserine exposure [[14], [15], [16]]. These platelets release abundant PMPs, further amplifying immune dysregulation.

By reframing platelets as immunomodulatory agents rather than passive targets, this review highlights novel avenues for therapeutic intervention. We suggest that targeting platelet activation, platelet–T cell interactions, and PMP release could complement or even surpass traditional strategies focused solely on suppressing autoreactive immune cells. A platelet-centric approach may offer more effective, tailored treatments for patients with chronic or refractory ITP and ultimately help restore immune homeostasis.

## 2 ITP as a heterogeneous autoimmune disorder

ITP is increasingly recognized as a clinically and immunologically heterogeneous disorder, rather than a single disease entity. Traditionally defined by isolated thrombocytopenia (<100 × 10^9^/L) in the absence of other causes, ITP comprises a diverse spectrum of clinical presentations, immune mechanisms, and treatment responses [17,18]. The heterogeneity of ITP is evident across several dimensions, including its etiology, immunopathogenesis, duration, and disease course.

ITP is broadly classified into primary ITP, which is idiopathic and not associated with an underlying condition, and secondary ITP, which occurs in the context of identifiable triggers or comorbidities [19]. Secondary forms are increasingly recognized and may be associated with systemic autoimmune diseases (e.g., systemic lupus erythematosus), chronic infections (e.g., HIV, HCV), lymphoproliferative disorders, certain medications, or immune dysregulation syndromes, including inborn errors of immunity (IEI) [19,20]. These genetic disorders, such as Cytotoxic T-Lymphocyte-Associated Protein 4 (CTLA-4) [21,22], haploinsufficiency [22,23], autoimmune lymphoproliferative syndrome (ALPS), and common variable immunodeficiency (CVID), offer insight into the complex immune dysfunctions that may underlie ITP and often feature refractory or atypical courses of thrombocytopenia [24,25].

In terms of disease duration, ITP is categorized as newly diagnosed (<3 months), persistent (3–12 months), or chronic (>12 months), with chronic ITP comprising the majority of adult cases [26,27]. Furthermore, a subset of patients experience refractory ITP, defined by failure to respond to multiple lines of therapy, including splenectomy [28]. This subgroup often reflects deeper immune dysregulation, with altered T and B cell homeostasis, impaired regulatory mechanisms, and resistance to conventional immunosuppression [29].

The immunopathogenic diversity in ITP further complicates its classification. While antiplatelet autoantibodies are a hallmark feature in many patients [30], others may lack detectable antibodies and instead show predominant T cell–mediated cytotoxicity [31], defective Treg function [32], or abnormal antigen presentation [33]. These distinctions are not only of academic interest—they carry potential diagnostic and therapeutic implications, as different immune mechanisms may respond variably to treatments such as corticosteroids, thrombopoietin receptor agonists (TPO-RAs), or emerging immunomodulatory agents.

Understanding and acknowledging the heterogeneity of ITP is critical for both clinical management and research. It provides the essential context for exploring emerging concepts in pathogenesis, such as the active immunomodulatory role of platelets, which may be especially relevant in chronic and refractory disease states where traditional models fall short.

## 3 Classical immune pathogenesis of ITP

The classical model of ITP centers on a loss of immune tolerance to platelet antigens, leading to autoimmune-mediated platelet destruction and impaired platelet production. This immune dysregulation is orchestrated by both humoral and cellular components, involving a complex interplay among B cells, T cells, plasma cells, dendritic cells, and macrophages [34].

A hallmark feature of ITP is the production of anti-platelet autoantibodies, primarily directed against glycoproteins on the platelet surface, including GPIIb/IIIa, GPIb/IX, and GPV [35]. These autoantibodies, mostly of the IgG isotype, opsonize platelets and mediate their clearance by Fcγ receptor–bearing phagocytes in the spleen and liver. Additionally, autoantibodies may interfere directly with megakaryocyte maturation and thrombopoiesis in the bone marrow, contributing to the observed reduction in platelet production [36].

Beyond antibody-mediated mechanisms, T cells play a central role in the pathogenesis of ITP. Autoreactive CD4^+^ Th cells promote B cell activation and antibody class switching, while cytotoxic CD8^+^ T cells have been shown to directly lyse platelets and megakaryocytes [9,37,38]. Importantly, patients with ITP often exhibit a Th1-polarized immune response alongside a reduction in Tregs, which are crucial for maintaining peripheral tolerance [39].

B cells are not only responsible for autoantibody production but also serve as antigen-presenting cells that can drive T cell activation and differentiation [40]. Long-lived autoreactive plasma cells, residing in bone marrow and other immune niches, contribute to disease chronicity and may persist despite B cell–depleting therapies such as rituximab [41,42]. The failure to eliminate these cells is increasingly recognized as a barrier to sustained remission.

Antigen-presenting cells, particularly dendritic cells (DCs) and splenic macrophages, further propagate the autoimmune response [43,44]. DCs from ITP patients display enhanced ability to present platelet antigens to autoreactive T cells, often in the context of heightened expression of costimulatory molecules and proinflammatory cytokines [44]. Additionally, monocyte-derived macrophages in the spleen mediate Fcγ receptor–dependent phagocytosis of opsonized platelets, completing the cycle of peripheral platelet destruction [35].

Taken together, these processes reflect a breakdown of central and peripheral tolerance in both the T and B cell compartments, exacerbated by dysregulated innate immune activation. While these mechanisms form the backbone of our current understanding of ITP, they do not fully explain the variability in clinical presentation and treatment response—thereby highlighting the need to explore additional pathways, including the active role of platelets as immune modulators, as discussed in subsequent sections.

## 4 Beyond hemostasis: platelets at the crossroads of coagulation and immunity

Platelets have been conventionally regarded as anucleate cell fragments primarily responsible for hemostasis and thrombosis. Their fundamental role in coagulation is well established, where they adhere to damaged endothelium, aggregate to form a hemostatic plug, and facilitate fibrin clot formation through the release of coagulation factors [45]. However, recent advances have expanded our understanding of platelets beyond their conventional function, and suggested their active participation in immune regulation and inflammatory processes [46].

Emerging evidence indicates that platelets are dynamic immune mediators capable of interacting with both innate and adaptive immune cells [7]. They express a range of immunomodulatory molecules on their surface, including CD40L, P-selectin, and Toll-like receptors (TLRs), which enable them to orchestrate immune responses [47]. Through their interactions with leukocytes, platelets can modulate inflammatory signaling pathways, promote neutrophil extracellular trap (NET) formation, and contribute to monocyte differentiation [[48], [49], [50]]. Furthermore, PMPs serve as potent carriers of cytokines, chemokines, and autoantigens, which promote immune activation in various inflammatory disorders.

**Fig. 1:**
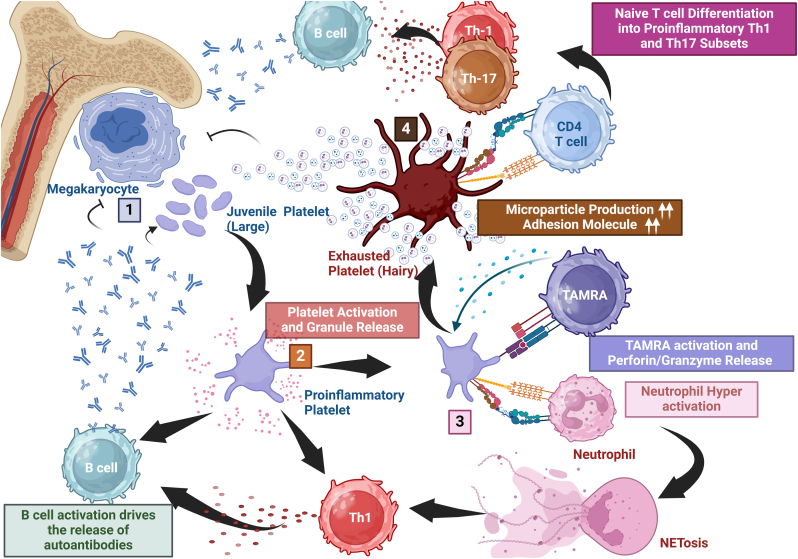
Platelet-Driven Immune Dysregulation in ITP Immature platelets originating from the bone marrow enter the circulation, where inflammatory factors and anti-platelet autoantibodies trigger their activation [1]. These activated, proinflammatory platelets release granules rich in mediators such as CD40L and TGF-β1, skewing T cell differentiation toward the Th1 phenotype and stimulating B cells to produce additional autoantibodies. This amplifies platelet destruction, suppresses platelet production, and perpetuates inflammation, thereby worsening ITP [2]. Activated platelets exhibit increased P-selectin expression and an active αIIbβ3 integrin conformation, enabling aggregation with leukocytes. Interaction with neutrophils promotes NETosis, fueling thromboinflammation, while aggregation with TEMRA cells induces perforin and granzyme release—processes that accelerate platelet destruction and drive platelets toward an exhausted state [3]. Exhausted platelets are functionally procoagulant platelets that are granule-depleted, expose phosphatidylserine (PS), and release large amounts of platelet microparticles (PMPs). Under microscopy, this same platelet phenotype may display surface projections, described morphologically as “hairy” platelets. Thus, “hairy” describes appearance, whereas “exhausted” denotes functional status—both refer to the same procoagulant, microparticle-releasing platelet subtype. PMPs, along with surface receptors, modulate immune cell activity, sustaining lymphocyte activation and promoting autoantibody production [4]. Moreover, PMPs and circulating autoantibodies impair megakaryocyte function, reducing platelet production and forcing the release of larger, immature platelets. This pathological loop perpetuates immune dysregulation and contributes to the chronicity of ITP. CD40L; CD40 ligand, ITP; Immune thrombocytopenia, PMPs; Platelet microparticles, TGF; Transforming growth factor, Th; T helper.

In ITP, the evolving role of platelets in immune regulation has become particularly evident [9]. Instead of being passive targets of immune destruction, platelets actively influence disease pathology. They engage in complex crosstalk with T cells through the P-selectin–PSGL-1 axis, modulating the dynamics of follicular Th and Tregs. This interaction disrupts immune tolerance, fueling autoimmunity and potentially amplifying autoantibody production [46]. Additionally, the presence of "exhausted" platelets, a distinct subpopulation with a procoagulant-like phenotype and the morphological features of "hairy" platelets, further underscores the immune-modulatory capacity of platelets in ITP. These platelets lose their coagulation function not only due to granule secretion but also through the shedding of GPVI (platelet glycoprotein VI, the primary collagen receptor), and the cleavage of the cytoplasmic domain of GPIIb/IIIa, rendering them hemostatically inactive [[51], [52], [53]]. Despite this, these hairy platelets actively drive immune dysregulation by producing PMPs, which fuel inflammatory cascades and perpetuate autoimmunity [54]. Fig. 1 illustrates some of the mechanisms by which platelets can drive immune dysregulation in ITP.

The recognition of platelets as active players in regulating immune responses has profound implications for understanding autoimmune diseases and developing targeted therapies. Strategies aimed at modulating platelet activation and platelet-mediated immune interactions may offer novel treatments, particularly in conditions like ITP, where platelet-immune dysregulation plays a central role in disease progression.

## 5 Platelets as immune regulators in ITP

ITP is traditionally characterized by autoantibody-mediated platelet destruction, yet emerging evidence suggests that platelets actively govern immune responses beyond being mere targets of immune attack [55]. As dynamic regulators of immunity, platelets engage in crosstalk with lymphocytes, modulating their activation, differentiation, and survival. These interactions are primarily mediated by platelet adhesion molecules, cytokine release, and microparticle generation, which can either promote immune tolerance or drive pathological inflammation [9,55,56].

### 5.1 Platelet activation and interaction with immune cells

In ITP, platelet activation is driven by autoimmune-mediated mechanisms that contribute to platelet destruction and heightened immune activity. Multiple pathways facilitate platelet activation, including Fc receptor signaling, complement activation, and direct platelet-immune cell crosstalk, all of which exacerbate the inflammatory and thrombocytopenic state found in this condition [57].

A central mechanism involves autoantibody-mediated platelet activation, where anti-platelet autoantibodies (e.g., against GPIIb/IIIa and GPIb-IX) bind to platelets, triggering FcγRIIA engagement [58,59]. This engagement initiates downstream signaling through Syk kinase (spleen tyrosine kinase) and PI3K/Akt pathways, promoting platelet degranulation, cytokine release, and surface exposure of P-selectin [59,60]. The expression of P-selectin facilitates platelet adhesion to PSGL-1-expressing T cells, reinforcing platelet-immune cell interactions and amplifying immune dysregulation in ITP.

### 5.2 PMPs and immune crosstalk

In addition to antibody-driven mechanisms, PMPs serve as crucial mediators of immune responses [55]. These microparticles are enriched in pro-inflammatory cytokines (IL-1β, CD40L, CXCL4) and immune-regulatory molecules, and can act as biological messengers influencing T cell activation, differentiation, and polarization [61]. PMPs contribute to Treg dysfunction, directly driving Th1 differentiation while indirectly promoting Th17 differentiation, thereby exacerbating immune dysregulation and platelet destruction [46,61,62]. Notably, PMPs contain a diverse range of pro-inflammatory and anti-inflammatory mediators, as well as mRNAs and microRNAs. The cargo composition of PMPs differs significantly in ITP patients compared to healthy individuals [55]. This altered molecular profile contributes to a heightened inflammatory state, explaining why increased PMP generation in ITP correlates with disease severity [55]. Additionally, PMPs interact with antigen-presenting cells (APCs), enhancing MHC-II expression and dendritic cell activation, thereby perpetuating autoreactive immune responses [63].

### 5.3 Exhausted "hairy" platelets and their role in PMP generation

A subpopulation of "exhausted" platelets with a distinct "hairy" morphology has been observed in ITP patients [64,65]. These platelets show cytoplasmic projections that may explain their increased propensity to generate PMPs. The increased release of PMPs further facilitates immune crosstalk, amplifying inflammatory and thrombo-inflammatory cascades [55]. This generation of hyperactivated PMPs not only promotes platelet-leukocyte crosstalk, but may also expose autoantigens, reinforcing immune dysregulation. Additionally, exhausted platelets exhibit activation of the NADPH oxidase (NOX) complex which not only increases intraplatelet ROS, but also amplifies the oxidative milieu, further exacerbating platelet hyperactivity and immune interactions [66].

Moreover, platelet mitochondrial dysfunction and oxidative stress drive two distinct pathways by which platelets can interact with immune cells. These are, firstly the direct binding of platelets via upregulated adhesion molecules, and secondly indirect modulation via calpain activation leading to enhanced microparticle formation [[67], [68], [69]]. The release of damage-associated molecular patterns (DAMPs), including mitochondrial DNA (mtDNA) and HMGB1, from activated platelets enhances inflammatory signaling via the TLR9 and NF-κB pathways, reinforcing the autoimmune cascade [70].

Together, these findings underscore the multifaceted role of platelet activation in ITP, suggesting that platelets are not only passive targets of immune destruction, but are also active mediators of immune dysfunction. Understanding these mechanisms may pave the way for novel therapeutic strategies that target platelet-immune interactions while preserving platelet hemostatic functions.

### 5.4 Platelet-derived factors and immune modulation

In ITP, platelets are not merely passive targets of immune attack but active participants in shaping immune responses. Upon activation, they release a repertoire of immunomodulatory factors—including P-selectin, TGF-β1, platelet factor 4 (PF4), and PMPs—that collectively influence immune cell activation, differentiation, and survival. These mediators contribute to a self-sustaining pro-inflammatory environment, exacerbating both platelet destruction and immune dysregulation (Table 1).

**Table 1:** Key mechanisms of platelet-driven immune dysregulation in ITP.

Interaction	Mechanism	Description	Implications in ITP
Direct Platelet-T cell Interaction (Cell-Cell contact)	Platelet activation drives aggregate formation with T cells.	Platelets interact with T cells via P-selectin/PSGL-1 and CD40/CD40L pathways, modulating T cell activation.	Promotes Th1/Th17 polarization, reduces Treg stability, and contributes to immune dysregulation.
	Platelet-Driven Antigen Presentation	Platelets express MHC class I and can cross-present antigens to cytotoxic T cells.	Supports immune surveillance but may exacerbate autoimmunity in ITP, leading to increased platelet destruction.
	Exhausted Platelets (due to defective clearance)	Impaired removal of apoptotic platelets leads to prolonged antigen exposure.	Enhances immune stimulation, contributing to persistent autoantibody production.
Indirect Platelet-T cell Interaction	Platelet-Derived Microparticles	PMPs carry pro-inflammatory cytokines, autoantigens, and danger signals.	Amplifies immune activation, enhances antigen presentation, and sustains autoimmunity.
	Anti-inflammatory Mediators Released by Platelets	Platelets release TGF-β, which regulates T cell differentiation.	Affects balance between Th17 and Tregs, influencing immune tolerance or inflammation.
	Inflammatory Mediators Released by Platelets	Platelets secrete cytokines such as IL-1β, IL-6, and CXCL4.	Exacerbates local and systemic inflammation, promoting sustained immune activation.

Among these, PF4 has emerged as a key modulator of T cell immunity. This CXC chemokine, released from platelet α-granules, interacts with CXCR3 on T cells to guide their migration and activation at inflammatory sites, promoting effector T cell accumulation [71,72]. In the context of ITP, PF4-mediated recruitment of T cells may enhance immune activation and facilitates platelet destruction.

Notably, Tan S., Li S. et al. [71] demonstrated that PF4 exerts profound effects on CD4^+^ effector memory T cells (Tem) via metabolic reprogramming. In vitro co-culture of Tem cells with platelets augmented both Th1 and Treg responses—transiently for Th1 cells and persistently for Tregs. Mechanistically, PF4 binding to CXCR3 attenuated Akt activity and reduced PGC1α phosphorylation, leading to increased TFAM expression and mitochondrial biogenesis. This metabolic shift elevated mitochondrial mass, oxygen consumption, ATP, and ROS production—thereby enhancing expression of T-bet in Th1 cells and FoxP3 in Tregs. Inhibition of mitochondrial function abrogated these effects, confirming the central role of the PF4–Akt–PGC1α–TFAM axis.

PF4 also influences T cell polarization, promoting differentiation into Th1 and Th17 subsets—both implicated in ITP pathogenesis. Through this mechanism, PF4 amplifies cytokine secretion and sustains chronic inflammation, positioning it as a double-edged sword in ITP and a promising therapeutic target.

In parallel, TGF-β1, predominantly released by platelets, plays a context-dependent role in immune modulation. When combined with IL-6, it promotes Th17 differentiation and inflammation, whereas in other settings it induces Treg development and immune tolerance [73,74]. TGF-β1 has also been shown to contribute to immune homeostasis through its interaction with myeloid-derived suppressor cells (MDSCs). Recent studies, such as that by Wang L. et al., have uncovered new facets of TGF-β1's role in ITP, particularly in the context of TPO receptor agonists (TPO-RAs). Platelet α-granules are rich in TGF-β1, which is crucial for the expansion and functional reprogramming of MDSCs.

In their study, Wang L. et al. [75] demonstrated that responders to TPO-RAs exhibited a parallel increase in both platelet and MDSC numbers. Using an active ITP murine model, they found that TPO-RAs enhanced the inhibitory activities of MDSCs. This was achieved through the arrest of plasma cell differentiation, reduction in Fas ligand expression on cytotoxic T cells, and the rebalancing of T-cell subsets. Mechanistically, transcriptome analysis revealed that the TGF-β/Smad signaling pathway was involved in the TPO-RA-induced expansion and suppression capacity of MDSCs, a process that was abolished by Smad2/3 knockdown. Importantly, platelet TGF-β1-deficient mice failed to show the TPO-RA-induced MDSC amplification, highlighting the crucial role of platelet-derived TGF-β1 in mediating this immune modulation.

Furthermore, the study by Wang L. et al. highlighted that patients with ITP who achieved a complete platelet response showed significantly better long-term outcomes than those with a partial response, underscoring the clinical relevance of platelet recovery in ITP management. These findings position platelet-derived TGF-β1 as a pivotal factor in re-establishing immune homeostasis, not only through its role in platelet regeneration but also via its ability to modulate immune cell interactions, including the reprogramming of MDSCs. This insight further emphasizes the complex interplay between platelets and immune cells in ITP pathogenesis, with platelet recovery playing a dual role in both restoring platelet count and promoting immune balance.

Another critical mediator is P-selectin, expressed on activated platelets, which binds PSGL-1 on T cells to facilitate platelet–T cell aggregate formation. This interaction enhances T cell activation but can also induce anergy and exhaustion, especially in CD4^+^ T cells, compromising their regulatory function [76]. Moreover, P-selectin–PSGL-1 binding downregulates FOXP3 in Tregs, reducing their numbers and contributing to the effector–Treg imbalance characteristic of ITP [46].

Together, these platelet-derived factors—particularly P-selectin and TGF-β1—form a complex immune signaling network that drives platelet–immune cell aggregation and perpetuates autoimmune responses in ITP. Understanding how these mediators influence immune cell interactions could open new therapeutic avenues, enabling the preservation of platelet hemostatic function while mitigating immune dysregulation. Importantly, these immune-modulating effects underscore the central role of platelets as active participants in immune orchestration. Their crosstalk with lymphocytes not only sustains chronic inflammation but also shapes T cell function and phenotype. This dynamic interaction paves the way for a deeper exploration of how platelets engage specifically with CD4^+^ and CD8^+^ T cells—interactions that drive the loss of immune tolerance, effector polarization, and T cell exhaustion characteristic of ITP, which we delve into in the following section.

## 6 Platelet-lymphocyte aggregation: the role of CD4^+^ and CD8^+^ T cells

### 6.1 CD4^+^ T cells and platelet interactions: immune priming and loss of tolerance

In ITP, CD4^+^ Th cells play a pivotal role in orchestrating immune responses and are crucial in platelet-immune interactions. Recent studies suggest that platelets, particularly when activated, are not merely passive entities but actively participate in the immune dysregulation that characterizes ITP [13,55]. The engagement of P-selectin on activated platelets with PSGL-1 on CD4^+^ T cells initiates a cascade of events that fuel immune-mediated platelet destruction [46].

Evidence from other inflammatory contexts supports this mechanism. In a study of patients undergoing on-pump coronary artery bypass grafting (CABG), Farhid et al. [77] demonstrated that acute inflammatory stress sharply increased platelet P-selectin expression, followed by a surge in PTCA formation. This interaction dynamically altered T cell populations, including transient CD4^+^ T cell depletion and marked shifts in regulatory Treg subsets. While CABG and ITP differ in etiology, these findings provide direct in vivo proof that platelet activation via P-selectin can modulate T cell differentiation and regulatory balance—a process likely amplified in ITP, where platelet activation is chronic and immune dysregulation persistent.

In the ITP setting, sustained platelet P-selectin expression and persistent PTCA formation may continuously skew CD4^+^ T cells toward Th1 and Th17 phenotypes, characterized by elevated IFN-γ and IL-17 production [78]. These cytokines are potent drivers of B cell activation, autoantibody generation, and tissue inflammation. Concomitantly, platelet-derived IL-1β and CD40L reinforce CD4^+^ T cell activation, forming a self-perpetuating loop that intensifies platelet destruction and inflammation [79]. CD40L further activates antigen-presenting cells (APCs), amplifying autoreactive T cell responses [80].

Beyond T cell modulation, platelet–neutrophil aggregation via the P-selectin–PSGL-1 axis promotes neutrophil extracellular trap (NET) formation, releasing damage-associated molecular patterns (DAMPs) such as HMGB1 [48,81]. This contributes to endothelial activation, pyroptosis, and further IL-1β release [[82], [83], [84]], propagating vascular and immune injury.

Importantly, the crosstalk between platelets and Tregs is significantly affected by this interaction. Platelet activation and its effects on Tregs suppress FOXP3 expression, thus impairing Treg regulatory function and intensifying immune dysregulation [46]. As a result, immune tolerance is lost, and autoreactive T cells, including CD4^+^ T cells, continue to drive inflammation. This process results in a vicious cycle of immune hyperactivity and platelet destruction, undermining the body's capacity to restore immune balance.

These findings further emphasize that platelets are active modulators of CD4^+^ T cell differentiation and function, shifting the immune response from tolerance to autoimmunity. Platelet-mediated Th1/Th17 skewing contributes significantly to the persistent inflammatory state observed in ITP, offering new avenues for therapeutic targeting.

### 6.2 Cytotoxic T cells and platelet interactions: T cell exhaustion and apoptosis

While CD8^+^ T cells have traditionally been associated with immune surveillance and pathogen clearance, their role in ITP has become increasingly evident [9]. CD8^+^ T cells normally carry out protective functions, but can undergo profound alterations in the setting of platelet-driven immune dysregulation [9,85]. In chronic inflammatory states like ITP, in addition to the platelets suffering exhaustion, T cells also exhibit an exhaustion phenotype, characterized by the upregulation of inhibitory receptors (PD-1, TIM-3, LAG-3), impairment of their cytotoxic function and persistent immune dysfunction, which all exacerbate disease progression [86].

Platelet-derived signals promote T cell exhaustion through PSGL-1-mediated aggregation, leading to CD8^+^ T cell death via either apoptosis or pyroptosis [86]. Paradoxically, this process depletes the cytotoxic T cell population while also maintaining a pro-inflammatory environment. In addition to pyroptosis, a highly inflammatory process that exacerbates inflammation [87], exhausted CD8^+^ T cells fail to clear autoreactive immune cells, allowing chronic inflammation to persist and perpetuate platelet destruction. This impairment of T cell cytotoxic function represents a key mechanism through which platelets influence immune homeostasis in ITP.

Insights from other chronic inflammatory conditions reinforce this paradigm. In HIV-1 infection, Wu F et al. [88] demonstrated that platelet–CD8^+^ T cell aggregates are markedly elevated in treatment-naïve individuals, correlating positively with viral load and negatively with CD4^+^ T cell count and CD4/CD8 ratio. These aggregates were associated with enhanced CD8^+^ T cell exhaustion and increased rates of both apoptosis and pyroptosis compared to unbound CD8^+^ T cells. Mechanistically, platelets induced caspase-1 activation in CD8^+^ T cells, which strongly correlated with plasma IL-1β and IL-18 levels, and platelet-derived cytokines remained elevated even after viral suppression.

Although the etiologies of HIV-1 and ITP differ, both conditions share the hallmark of sustained immune activation. By analogy, persistent platelet–CD8^+^ T cell aggregation in ITP likely drives caspase-1–dependent pyroptosis, depleting cytotoxic T cells while perpetuating a cytokine-rich inflammatory environment. This dual effect—loss of immune surveillance alongside amplification of inflammation—represents a critical mechanism by which platelets exacerbate immune dysregulation.

Moreover, platelet–T cell interactions maintain CD8^+^ T cells in a partially exhausted state through continuous cytokine release and P-selectin–PSGL-1 engagement. This biphasic dynamic, transitioning from initial hyperactivation to chronic exhaustion, underscores the reciprocal and self-reinforcing interplay between platelets and cytotoxic T cells. Understanding these mechanisms reveals actionable therapeutic targets, including inhibition of PSGL-1/P-selectin interactions and blockade of caspase-1–mediated pyroptosis, offering potential strategies to restore CD8^+^ T cell functionality and disrupt persistent inflammation in ITP.

## 7 Future therapeutic directions

**Fig. 2:**
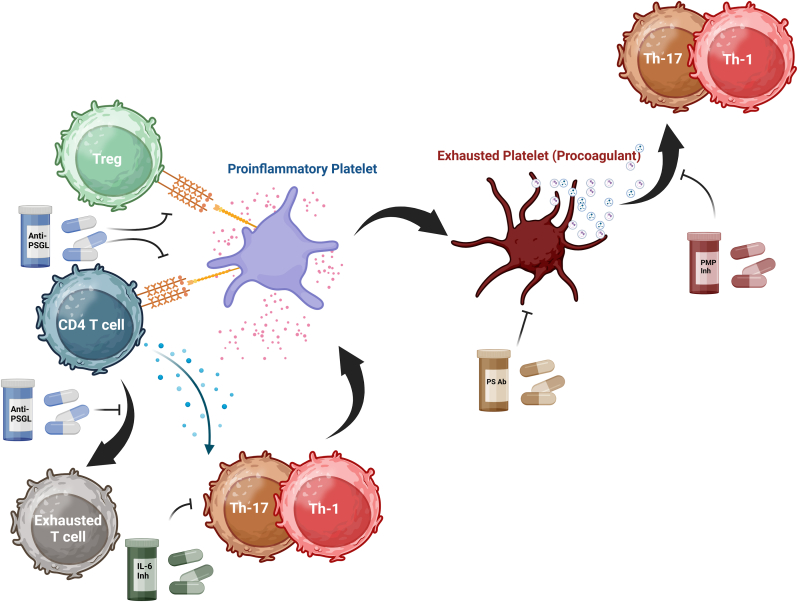
Targeting Platelet-Driven Immune Dysregulation in ITP: Therapeutic Strategies Multiple targeted strategies have been proposed to counteract the pathogenic role of platelets in ITP. Since platelet–leukocyte aggregation is largely mediated by the P-selectin–PSGL-1 axis, blocking this interaction with an anti-PSGL-1 antibody could prevent platelet–Treg aggregation, preserving Treg function and preventing their decline. This could reduce systemic inflammation and restore immune balance. Inhibition of the P-selectin–PSGL-1 axis may also modulate CD4^+^ T cell differentiation, reducing Th1 and Th17 polarization while restoring a balanced Th17/Treg ratio. Anti-PSGL-1 therapy could additionally mitigate T cell exhaustion and broader immune dysregulation, offering a targeted approach to immune reprogramming. The inflammatory milieu in ITP also fosters the formation of exhausted platelets—procoagulant platelets that are granule-depleted, PS-exposing, and release abundant PMPs. Morphologically, these same exhausted platelets may appear “hairy” under microscopy, a descriptive term that refers to their surface projections. Thus, “hairy” and “exhausted” denote the same platelet subtype from morphological and functional perspectives, respectively. These dysfunctional platelets release PMPs and express high levels of adhesion molecules, amplifying inflammation and immune activation. Targeting PS with specific antibodies could accelerate the clearance of these platelets, limiting their pathological effects. In parallel, PMP-targeted therapies could modulate their production and function, disrupting the pro-inflammatory and pro-thrombotic cycles that sustain ITP pathology and offering a refined, durable therapeutic strategy. ITP; Immune thrombocytopenia, PMPs; Platelet microparicles, PSGL-1; P-Selectin Glycoprotein Ligand 1, Th; T helper.

The intricate interplay between platelets and T cells in ITP is more than an epiphenomenon, it is a driving force behind chronic immune dysregulation. Because platelets act as both targets and mediators of immune activation, their interactions with CD4^+^ and CD8^+^ T cells can perpetuate a self-sustaining cycle of inflammation, autoantibody production, and platelet destruction [55]. Understanding the molecular crosstalk between these two cellular populations opens new therapeutic possibilities, allowing for the development of targeted interventions that go beyond conventional immunosuppressive strategies. Fig. 2 illustrates some possible therapeutic approaches that could be used to restore the balance of the immune system in ITP.

### 7.1 Targeting Platelet-T cell interactions in ITP therapy

One of the most promising therapeutic strategies in ITP involves disrupting platelet-T cell crosstalk, a key driver of chronic immune activation. One of the most harmful interactions is the P-selectin–PSGL-1 axis, which mediates platelet adhesion to T cells and prolongs immune activation [89]. CD4^+^ T cells, and particularly Tregs, express a high-affinity form of PSGL-1, making them especially susceptible to long term platelet binding. This sustained interaction downregulates FOXP3 expression, weakening Treg-mediated immune tolerance and tipping the balance toward a Th1/Th17-driven inflammatory state [46]. Additionally, platelet-T cell aggregates promote T cell receptor (TCR) signaling, leading to prolonged T cell activation and exacerbated autoimmune responses.

To disrupt these pathological interactions, several targeted therapeutic strategies are under investigation. P-selectin inhibitors, such as crizanlizumab, have demonstrated some efficacy in reducing platelet adhesion in other inflammatory diseases, suggesting their potential to limit platelet-T cell aggregation and mitigate chronic immune activation in ITP [90,91]. However, despite its promise, this agent may exacerbate hemorrhagic events and cause bleeding, highlighting the need for careful balancing of therapeutic benefits with safety concerns in treating ITP. PSGL-1 blockade could be another promising avenue, because modulating PSGL-1 interactions might help to preserve Treg function, restore immune balance, and prevent unchecked Th1/Th17 activation. Another emerging area of interest is targeting PMPs, the most abundant extracellular vesicles in the circulation [92]. These PMPs serve as carriers of autoantigens and pro-inflammatory cytokines, fueling autoantibody production and immune cell activation. Strategies aimed at reducing PMP generation or neutralizing their immune-stimulating cargo may help to attenuate immune activation and disease progression. However, further research is needed to determine the safety and efficacy of such interventions in ITP patients.

### 7.2 Possible modulation of PSGL-1 pathways to restore immune balance

The PSGL-1 signaling pathway plays a paradoxical role in ITP pathogenesis, functioning as both an immune checkpoint and a driver of immune dysfunction. While PSGL-1 binding to P-selectin has been implicated in T cell exhaustion and anergy, this same interaction also contributes to FOXP3 downregulation, leading to Treg destabilization and increased autoreactivity [46,76]. A deeper understanding of these dual roles of PSGL-1 in T cell function is critical for designing more selective therapeutic interventions. One of the primary goals of PSGL-1 modulation is to restore Treg function while simultaneously preventing excessive immune activation. Targeted approaches that stabilize FOXP3 expression and preserve PSGL-1-mediated immune tolerance could help rebalance T cell responses and prevent further platelet destruction. Another important consideration is to prevent T cell exhaustion, because chronic platelet engagement leads to upregulation of PD-1, TIM-3, and LAG-3, which are markers of dysfunctional, non-responsive T cells. By selectively modulating PSGL-1 interactions, it may be possible to reinvigorate exhausted T cells, allowing them to regain immunoregulatory function while preventing overactivation. Additionally, modulating PSGL-1 pathways could help to rebalance Th1/Th17 polarization, as both of these pro-inflammatory subsets express high levels of PSGL-1. Shifting the immune response toward a less inflammatory, more tolerogenic state could help control autoreactive T cell activity, reduce cytokine-driven platelet destruction, and promote long-term immune stability. The ability to fine-tune T cell responses while preserving immune homeostasis could be particularly beneficial for patients with chronic or refractory ITP, who often suffer repeated relapses despite standard-of-care therapy. However, blocking the P-selectin–PSGL-1 axis could interfere with leukocyte adherence to the endothelium and suppress migration, potentially putting patients at an increased risk of infections.

### 7.3 Next-generation therapeutics

As our understanding of platelet-driven immune dysregulation continues to evolve, next-generation therapeutic approaches must move beyond traditional broad-spectrum immunosuppressants and toward precision-based immune modulation. One critical focus for these approaches is the accelerated removal of exhausted platelets, which are characterized by increased PS expression on their surface. These exhausted platelets play a pivotal role in exacerbating inflammation and perpetuating ITP, both through direct interactions with leukocytes, particularly T cells, and through the release of DAMPs and PMPs [7,93]. Their persistence in the circulation contributes to ongoing immune dysregulation, making their targeted clearance a key therapeutic strategy.

Several innovative strategies are currently under investigation. One promising approach is dual-modulation therapy, where P-selectin or PSGL-1 inhibitors are combined with cytokine-modulating agents, such as IL-6 inhibitors, or immune checkpoint receptor inhibitors like VISTA (V-domain Ig suppressor of T cell activation) to more effectively reshape immune responses [94]. Another emerging therapeutic approach is PMP-targeted therapy, which aims to limit PMP release or neutralize the pro-inflammatory contents, thereby reducing antigenic stimulation of autoreactive B and T cells.

Moreover, strategies to accelerate the removal of exhausted platelets could be used to prevent their prolonged interaction with immune cells. Drugs such as anti-PS antibodies or agents that enhance platelet clearance by macrophages or the reticuloendothelial system, could help to selectively remove these dysfunctional platelets. Novel approaches targeting PS expression on platelets could also enhance the recognition of these cells by phagocytes, thereby reducing their accumulation and related inflammatory responses.

The future of ITP treatment is expected to lie in precision immunotherapy, where platelet-driven immune dysregulation is targeted at its core, rather than broad immunosuppression. Modulating platelet-T cell interactions, intervening in the PSGL-1 axis, reducing PMP-driven inflammation, and accelerating the clearance of exhausted platelets could be critical steps in developing more effective, long-term ITP control strategies. By integrating hematology, immunology, and vascular biology, next-generation therapies have the potential to revolutionize ITP management, providing durable remissions with fewer side effects. A multidisciplinary approach will be essential to design novel, patient-specific regimens that minimize immune dysregulation while preserving immune function.

## 8 Discussion

The intricate interplay between platelets and T cells in ITP has redefined our understanding of immune dysregulation in this disease. Far from being passive bystanders, platelets actively contribute to chronic inflammation, immune activation, and persistent platelet destruction. This review highlights the key mechanisms underlying platelet-driven immune dysfunction, including platelet-T cell interactions, PMPs as autoimmune triggers, and defective platelet clearance. These findings challenge the conventional view of ITP as a purely antibody-mediated disorder and emphasize the central role of platelets as immune modulators.

### 8.1 Unanswered questions and future research directions

Despite growing evidence in favor of platelet-driven immune dysregulation, several critical knowledge gaps remain. One key question is how platelet-derived TGF-β influences the balance between pro-inflammatory Th17 cells and immunosuppressive Tregs. While TGF-β can drive both immune tolerance and inflammation, its role in ITP remains poorly understood. Additionally, the PSGL-1 signaling pathway, which mediates platelet-T cell interactions, appears to exert dual effects on immune regulation, promoting T cell exhaustion while simultaneously destabilizing Tregs. Future studies should clarify whether modulating PSGL-1 interactions could selectively restore Treg function while preventing T cell exhaustion.

Another major knowledge gap is the role of PMPs in sustaining autoimmune responses. As the most abundant extracellular vesicles in the circulation, PMPs transport pro-inflammatory cytokines and autoantigens, potentially amplifying T cell and B cell activation. However, it remains unclear whether PMPs directly contribute to autoantibody production or simply act as secondary immune amplifiers. Addressing these uncertainties will be critical for developing more targeted treatments for ITP.

Finally, individual patient factors, such as genetic predisposition, comorbidities, and environmental influences, may shape platelet-immune interactions in ways that remain largely unexplored. Identifying biomarkers that predict disease severity or treatment response could pave the way for personalized, precision-based ITP therapies.

### 8.2 Clinical implications and therapeutic outlook

The growing recognition of platelets as active immune regulators has opened new therapeutic avenues beyond conventional broad-spectrum immunosuppression. Targeting platelet-T cell interactions could interrupt the cycle of chronic immune activation, offering a more specific and durable treatment strategy. One promising approach is blocking the P-selectin–PSGL-1 axis, which plays a key role in platelet-driven immune activation. Inhibiting this pathway may reduce platelet-T cell aggregation, limit Th1/Th17 polarization, and restore Treg function, potentially ameliorating autoimmunity without the need for global immune suppression.

Another emerging strategy involves modulating PMP release. Since PMPs carry autoantigens and inflammatory mediators, limiting their production or neutralizing their pro-inflammatory cargo could help dampen immune hyperactivation. Additionally, therapies aimed at enhancing apoptotic platelet clearance could prevent excessive antigen exposure, reducing immune stimulation and autoantibody production.

Beyond platelet-targeted treatments, approaches that rebalance Th1/Th17 polarization and restore Treg function are gaining attention. Small molecules or biologics that stabilize FOXP3 expression in Tregs could reinforce immune tolerance, while IL-6 inhibitors or TGF-β modulators may limit pathological Th17 expansion. These strategies offer a more precise immunomodulatory approach, preserving immune homeostasis while mitigating disease severity.

## 9 Conclusion

By redefining the role of platelets in immune pathology, this review challenges the traditional emphasis on autoantibodies as the sole drivers of ITP. Instead, it presents a more nuanced view of ITP as a disease in which platelets play a central role in orchestrating immune dysregulation. As we move toward next-generation immunomodulatory therapies, targeting platelet-immune interactions will be at the forefront of advancing ITP treatment—offering durable remission and improved quality of life for patients suffering from chronic and refractory disease.

A multidisciplinary approach integrating hematology, immunology, and vascular biology will be essential in the design of precision-based interventions that provide long-term disease control while preserving immune function. The future of ITP management lies in leveraging cutting-edge immunotherapy approaches to not only control platelet destruction but to restore immune balance as a whole.

## CRediT authorship contribution statement

**Fatemeh Farhid:** Writing – original draft. **Hadi Rezaeeyan:** Writing – original draft. **Reza Habibi:** Writing – original draft. **Ehsan Kamali Yazdi:** Writing – original draft. **Michael R. Hamblin:** Writing – original draft. **Jalal Naghinezhad:** Writing – original draft.

## Consent for publication

Not applicable.

## Funding

None.

**Table:** Table of Abbreviations

Abbreviation	Full Term
ITP	Immune Thrombocytopenia
TGF-β1	Transforming Growth Factor Beta 1
PF4	Platelet Factor 4
PMPs	Platelet-Derived Microvesicles
TPO-RAs	Thrombopoietin Receptor Agonists
MDSCs	Myeloid-Derived Suppressor Cells
CXCR3	C-X-C Chemokine Receptor Type 3
T-bet	T-box Transcription Factor
FoxP3	Forkhead Box P3
TFAM	Transcription Factor A, Mitochondrial
PGC1α	Peroxisome Proliferator-Activated Receptor Gamma Coactivator 1 Alpha
Th	T Helper
PD-1	Programmed Cell Death Protein 1
Tregs	Regulatory T Cells
IL	Interleukin
TIM-3	T-cell Immunoglobulin and Mucin Domain 3
LAG-3	Lymphocyte-Activation Gene 3
TCR	T Cell Receptor
PS	Phosphatidylserine
PSGL-1	P-selectin Glycoprotein Ligand 1
VISTA	V-domain Ig Suppressor of T Cell Activation
DAMPs	Damage-Associated Molecular Patterns
NETs	Neutrophil Extracellular Traps
TPO	Thrombopoietin

## Declaration of competing interest

The authors declare that they have no conflict of interest.

## Data Availability

This is a review study, and it is not an original. Data availability is corresponding author responsibility.
